# Bacterial Exopolysaccharides from Extreme Marine Habitats: Production, Characterization and Biological Activities

**DOI:** 10.3390/md8061779

**Published:** 2010-06-03

**Authors:** Annarita Poli, Gianluca Anzelmo, Barbara Nicolaus

**Affiliations:** 1 Institute of Biomolecular Chemistry, C.N.R., Via Campi Flegrei 34, 80078 Pozzuoli, Naples, Italy; E-Mail: annarita.poli@icb.cnr.it(A.P.); 2 Department of Environmental Sciences, Parthenope University of Naples, Centro Direzionale, Isola C4, (80143) Naples, Italy; E-Mail: gianluca.anzelmo@uniparthenope.it(G.A.)

**Keywords:** chemical composition, exopolysaccharides, extremophiles, marine bacteria, biological activity

## Abstract

Many marine bacteria produce exopolysaccharides (EPS) as a strategy for growth, adhering to solid surfaces, and to survive adverse conditions. There is growing interest in isolating new EPS producing bacteria from marine environments, particularly from extreme marine environments such as deep-sea hydrothermal vents characterized by high pressure and temperature and heavy metal presence. Marine EPS-producing microorganisms have been also isolated from several extreme niches such as the cold marine environments typically of Arctic and Antarctic sea ice, characterized by low temperature and low nutrient concentration, and the hypersaline marine environment found in a wide variety of aquatic and terrestrial ecosystems such as salt lakes and salterns. Most of their EPSs are heteropolysaccharides containing three or four different monosaccharides arranged in groups of 10 or less to form the repeating units. These polymers are often linear with an average molecular weight ranging from 1 × 10^5^ to 3 × 10^5^ Da. Some EPS are neutral macromolecules, but the majority of them are polyanionic for the presence of uronic acids or ketal-linked pyruvate or inorganic residues such as phosphate or sulfate. EPSs, forming a layer surrounding the cell, provide an effective protection against high or low temperature and salinity, or against possible predators. By examining their structure and chemical-physical characteristics it is possible to gain insight into their commercial application, and they are employed in several industries. Indeed EPSs produced by microorganisms from extreme habitats show biotechnological promise ranging from pharmaceutical industries, for their immunomodulatory and antiviral effects, bone regeneration and cicatrizing capacity, to food-processing industries for their peculiar gelling and thickening properties. Moreover, some EPSs are employed as biosurfactants and in detoxification mechanisms of petrochemical oil-polluted areas. The aim of this paper is to give an overview of current knowledge on EPSs produced by marine bacteria including symbiotic marine EPS-producing bacteria isolated from some marine annelid worms that live in extreme niches.

## 1. Introduction

Exopolysaccharides (EPSs) are high molecular weight carbohydrate polymers that make up a substantial component of the extracellular polymers surrounding most microbial cells in the marine environment. They constitute a large fraction of the reduced carbon reservoir in the ocean and enhance the survival of marine bacteria influencing the physicochemical environment in proximity of the bacterial cell. Moreover, they assist the microbial communities to endure extremes of temperature, salinity, and nutrient availability. In recent years the increased demand for natural polymers for pharmaceutical, food and other industrial applications has led to a remarkable interest in polysaccharides produced by microorganisms. Indeed, a substantial interest has aroused with regard to the isolation and identification of new microbial polysaccharides that might have innovative applications as gelling, emulsifier and stabilizer agents [1]. These biopolymers have emerged as new polymeric materials with novel and unique physical characteristics and therefore they have found extensive applications. Many microorganisms (many species of Gram-positive and Gram-negative Bacteria, Archaea, Fungi and some Alga) are known to produce extracellular polysaccharides or exopolysaccharides (EPS). This term was used for the first time by Sutherland in 1972 [2] in order to describe carbohydrate polymers produced by marine bacteria with high molecular weight. The advantages of microbial EPSs with regard to plants or marine macroalgal polymers are related to their chemical and physical properties and they embody a stable supply as well [3]. As far as the microbial biodiversity is concerned, bacterial EPSs exhibit a wide range of chemical structures: several EPSs display a high molecular weight as well as a heteropolymeric composition. Bacterial EPS usually occur in two forms: as capsular polysaccharides (CPS) where the polymers are covalently bound to the cell surface, and as slime polysaccharides which either remain attached (loosely bound) to the cell surface or are found in the extracellular medium as amorphous matrix [4,5].

In their natural environment, most bacteria occur in microbial aggregates whose structural and functional integrity is based on the presence of a matrix of extracellular polymeric substances and the EPS production seems to be essential for their survival [6]. In particular, marine polysaccharides, together with other macromolecules such as proteins, lipids and nucleic acids, comprise the organic matrix present in the intracellular space of microbial biofilms, which represents one of the largest reservoirs of reduced carbon on earth [6,7]. Moreover, the recent focus on extreme marine habitat has raised increasing attention on bacteria thriving in these conditions: extremophiles. These organisms, adopting special metabolic pathways and protective mechanisms to survive, represent a model to study the stability and the possibleroles of their biomolecules.

## 2. Roles of Microbial EPS in the Marine Environment

The physiological role of EPS depends on the ecological niches and the natural environment in which microorganisms have been isolated as well. Indeed, the EPS production is a process that requires a noticeable energy cost of up to 70%, representing a significant carbon investment for microorganisms. However, the benefits related to EPSs are significantly higher than costs considering the increasing growth and survival of microorganisms in their presence [8]. Indubitably, they possess a protective nature: the EPSs, forming a layer surrounding a cell, provide an effective protection against high or low temperature and salinity or against possible predators. They are essential in the aggregate formation, in the mechanism of adhesion to surfaces and to other organisms, in the formation of biofilm and in the uptake of nutrients [1,9–12]. In particular, studies of sea ice microbial communities have also found bacteria strongly associated to particles and have pointed out that microbial EPS played an important role in cryoprotection [13,14]. Moreover, the rate of synthesis and the amount of exopolysaccaride accumulated as capsular form in pathogenic bacteria influence their pathogenicity, in that capsular polysaccharides do not stimulate the immune system for their ability to mimic the cell surface of host cells.

EPSs display an important role in biofilm matrix in regard to the biochemical interactions between bacteria and surrounding cells [5,15]. The hydrated biofilms offer a stable micro-environment in which extracellular enzymes can find storage and in the same time facilitating cellular uptake of small molecules [11]. In addition, in a natural aquatic environment, the nutrients can interact with exopolymers in order to increase the rate of substance uptake and concentrate dissolved organic compounds, making them available to support microbial growth [5,15]. Finally, it has been proved that strains isolated from deep-sea hydrothermal vents show resistance to heavy metals and their purified EPSs presented the capacity to bind metals and toxic substances [16–18]. Indeed, these exopolymers exhibit a polyanionic state in marine environment displaying a high binding affinity for cations as well as trace metals. Since they generally contain uronic acids with a percentage between 20–50% of the total polysaccharide content, the acidic carboxyl groups attached are ionizable at seawater pH. Furthermore, EPS produced by some Antarctic bacterial isolates contain uronic acids and sulfate groups and may act as ligands for cations present as trace metals in the Southern Ocean environment, enhancing the primary production of microbial communities usually limited by poor availability of trace metals such as iron (Fe^+3^) [19].

## 3. Structure and Production of EPS by Marine Bacteria

Most EPSs produced by marine bacteria are heteropolysaccharides containing three or four different monosaccharides arranged in groups of 10 or less to form repeating units [5]. Components most commonly found in marine EPS are monosaccharide such as pentoses (as D-arabinose, D-Ribose, D-Xylose), hexoses (D-Glucose, D-Galactose, D-Mannose, D-Allose, L-Rhamnose, L-Fucose), amino sugars (D-Glucosamine and D-Galactosamine) or uronic acids (D-Glucuronic acids, D-Galacturonic acids). Organic or inorganic substituents such as sulfate, phosphate, acetic acid, succinic acid and pyruvic acid may also be present [20]. Most polymers are linear, with an average molecular weight ranging from 1 × 10^5^ to 3 × 10^5^ Da [21]. Some EPS are neutral macromolecules, but the majority of them are polyanionic for the presence of uronic acids or ketal-linked pyruvate or inorganic residues such as phosphate or sulfate as well [22]. Moreover, the linkages between monosaccharides that have been most commonly found are 1,4-β- or 1,3-β- linkages in the backbones characterized by strong rigidity and 1,2-α- or 1,6-α-linkages in the more flexible ones. The physical properties of polysaccharides are deeply influenced by the way the monosaccharides are arranged together and the assemblage of the single polymer chains [23]. It has been widely accepted that many EPSs undergo transition from an ordered state, especially at low temperature or in the presence of some ions, to a less ordered state at high temperature or in absence of ions, enabling the conversion of extracellular matrices from a gel to a solid state. For instance, solutions of the EPS produced by *Alteromonas* strain 1644, originated from a polychaete annelid living in the proximity of deep-sea hydrothermal vents, displayed very low viscosity values at low ionic concentration (below 0.03 M) [24]. As a result, it formed a gel at higher ionic concentrations or exhibited an unusual high temperature-dependent viscosity in solution at low polymer concentration. This behavior could also depend on the nature of the ions. In addition, *Alteromonas* strain 1644 is able to produce two kind of EPS, and this a very peculiar microbial characteristic, as only a few reports are known about simultaneous production of two different EPSs by the same microorganism [25]. This may be attributed to the difficulty in separating possible mixtures of exopolysaccharides but, in this case, the separation of the two polysaccharides produced by strain 1644 has been greatly facilitated by their different gelling properties.

Since the analysis of the extracellular matrix polysaccharides in the natural marine environment is difficult due to the low abundance of each polymer, the possibility to grow a single isolated strain under controlled laboratory conditions represents a suitable approach to investigate microbial EPS production [19]. However, there is no single set of culture conditions that guarantees high EPS yields since microorganisms differ in the critical factors for maximum EPS production: carbon and nitrogen source utilization, mineral requirements, temperature and optimal pH. Moreover, it is possible to modulate the molecular mass, the number of residues and the degree of branching of EPS by using a physiological control. Indeed, the nutritional and environmental conditions (culture conditions) can affect the yield and quality of microbial EPS [26]. Sutherland [4] showed that the EPS production increased if marine bacteria were grown in laboratory conditions on limited nutrients (such as nitrogen, phosphorus, sulfur and potassium). In general, suboptimal temperature of growth, osmotic stress or other physical factors that restrict the growth may enhance the EPS production. Moreover, the choice of selected carbon source (sugar or non-sugar sources) in the growth medium represents the first step for the optimization of EPS production. Actually, *Hahella chejuensis*, a microorganism isolated from marine sediment collected from Marado, Cheju Island, Republic of Korea, produced the highest EPS yield in a growth media emended with sucrose [27,28] while acetate was the most efficient carbon source for EPS production in *Halomonas alkaliantarctica*, strain CRSS, an haloalkalophilic bacteria isolated from saline lake in Antarctica [29,30]. In particular, strain CRSS was able to synthesize EPSs with different chemical composition on various substrates utilized as nutrients: a mannan or a xylo-mannan was produced in two different complex media and a fructo-glucan when the strain was grown on minimal media containing acetate as sole carbon source [29]. This is a common occurrence described for other strains, since culture conditions can modify the yield and the structure of polymers [31]. In regard to the nitrogen source, in general for EPS production, ammonium sulfate, peptone, sodium nitrate, urea and yeast extract are the components most currently used. In general, the presence of an organic nitrogen source promotes both the growth rate and the EPS production [32], even if there is some evidence showing that EPS production was higher at lower nitrogen concentration as suggested by Gorret *et al.* [33]. Samain *et al.* [24] used the fed-batch method in order to improve the EPS yield by an *Alteromonas* strain 1644: they controlled the growth by substituting the complex medium with a mineral defined medium containing ammonium chloride as a sole nitrogen source (nitrogen limitation). The initial ammonium chloride concentration was 0.4 g/L, and after the initial exponential phase, an ammonium chloride solution was continuously fed into the medium in order to maintain a small level of protein synthesis. During the entire feeding phase, the ammonium did not accumulate indicating that the growth was nitrogen-limited. In these conditions, EPS production started as soon as the ammonium had been depleted and continued almost linearly until the end of the fermentation, reaching a yield of 50% higher than obtained previously with the complex medium.

Sutherland [4] stated that there is a competition between EPS and cell-wall polymer (lipopolysaccharides, peptidoglycan, *etc.*) biosynthesis since the isoprenoid glycosyl lipid carriers and, consequently, EPS production, is not growth-associated. Recently, it has been found that most bacteria release the highest quantity of EPS in the stationary growth phase: *Alteromonas* strain 1644 [24], strain 4004, a thermophilic bacterium belonging to *Geobacillus* genus isolated from sea sand in Ischia Island (Italy) [34] and strain B3-15, isolated from marine hot spring at Vulcano Island, classified as a new strain of *Bacillus licheniformis* [35], are examples of microorganisms that produce high amounts of EPS in this phase. However, there are microorganisms that produce the maximum EPS during the exponential growth phase as reported for *Pseudoalteromonas antarctica* strain NF3 isolated from a glacial marine sludge at the South Shetland Island in Antarctica [36]. In *Alteromonas macleodii* subsp. *fijiensis*, isolated from a deep sea vent, the production of EPS began at the end of the exponential phase and continued throughout the stationary phase, reaching 6 g/L at 60 h of incubation [37].

Another shifting parameter during the EPS production is the rheology of the growth media: the broth develops non-Newtonian characteristics acting as a pseudoplastic fluid where the viscosity decreases with increasing shear rate. This change can be caused by the presence of EPS and their metabolic products, as well as the lack of homogeneity in terms of mixing, mass and oxygen. In these conditions, the EPS producer bacteria, exposed to such variable gradients, can produce heterogeneous EPS in terms of molecular weight, branching capacity, *etc.* The rheological shifts during the fermentation process could be used as a parameter to monitor the constancy of EPS quality and production [38]. Indeed, the aeration rate could be a parameter that affects EPS production as well. Lee *et al.* [28] reported that high aeration rates generally enhanced EPS production and increased the viscosity of the culture broth regarding *Hahella chejuensis*. Moreover, the use of some detergents such as Tween 40 (polyoxyethylene sorbital monopalmitate), Tween 80 (polyoxyethylene sorbital monooleate), CHAPS (3-[(3-cholamidopropyl) dimethyl ammonio]-1-hydroxypropane-sulfonate) and Triton X 100 (nonaethylene glycol octylphenol ether) may ameliorate oxygen concentration in the growth media, increasing the production of EPS [38].

## 4. Marine EPS-Producing Microorganisms Isolated from

### 4.1. Deep-Sea Hydrothermal Vents, Volcanic and Hydrothermal Marine Areas, Shallow Submarine Thermal Springs

To date, mainly mesophilic heterotrophic bacteria have been investigated rather than thermophilic microorganisms, even though the latter microorganisms possess thermostable enzymes with interesting biotechnological application for large scale industrial production. Actually, although considerable information related to the chemical composition, the rheological properties and metal binding capability have been determined, only a few polymers have been fully characterized so far.

**Figure 1:**
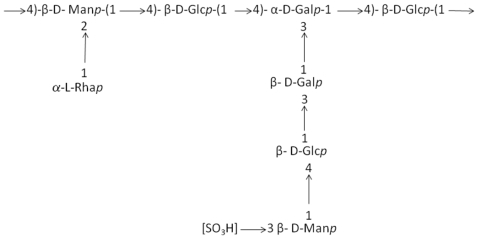
The repeating unit of EPS secreted by *Pseudoalteromonas* strain 721 [39].

Deep-sea hydrothermal vents, volcanic and hydrothermal marine areas, and shallow submarine thermal springs offer a new source of EPS producer bacteria (Table 1). *Pseudoalteromonas* strain 721 produced an EPS containing an octasaccharide repeating unit with two side chains (Figure 1), [39,40]. This EPS exhibited gel formation and viscoelastic behavior at increasing temperature.

*Alteromonas macleodii* subsp. *fijiensis* is an aerobic, mesophilic bacterium isolated from a hydrothermal vent at a depth of 2600 m in a rift system of the North Fiji basin [37,41]. This strain produced an EPS with an high metal-binding maximum capacity of up to 316 mg Pb(II)/g polymer [16,17]. The repeating unit consists of an hexasaccharide containing three uronosyl residues with a branch point at a galacturonosyl residue and a side chain terminated by a 4,6-*O*-(1-carboxyethylidene)-β-D-Man*p* [41]. Proposed uses for this polymer include water treatment and removal of heavy metal pollutants. Moreover, the xanthan-like EPS produced by *Alteromonas macleodii* subsp. *fijiensis* could find application as a food-thickening agent [41]. In addition, experiments conducted with this EPS encouraged its use in the bone healing application as it showed ability to promote the adhesion of rat calvaria osteoblastic cells *in vivo* [42]. Additionally, it could also be used in cardiovascular diseases as suggested by Colliec Jouault *et al.* [43].

Indeed, a valuable source of EPS is embodied by bacteria isolated from volcanic and hydrothermal marine areas and, in particular, some thermophiles, including *Methanosarcina*, *Haloferax*, *Haloarcula*, *Sulfolobus* and *Bacillus* species, and more recently *Thermotoga marittima* and *Thermococcus litoralis* have been studied so far as producers of unusual extra-cellular polysaccharides [35,44]. In particular, *Thermococcus litoralis* has been showed to produce EPS in a sulfur-free, defined growth medium with growth rates and cell yields comparable to those obtained on complex media. This microorganism is a heterotrophic facultative sulfur-dependent hyperthermophilic Archaeon, isolated from a shallow submarine thermal spring with an optimal growth temperature of 88 °C. Although extracellular polysaccharides have been identified in several marine extreme environments, the production of polysaccharides by extremophiles has been investigated only to a minor extent. The EPS produced by *T. litoralis* contains mannose as the only monosaccharidic constituent and this is a very peculiar feature for a prokaryote since the production of mannan-like constituents are typically produced by eukaryotes such as plants or yeasts [45]. According to the initial dry weight of the material, 1 to 2% sulfate and 1.5 to 4.5% phosphorus were also found in *T. litoralis* EPS. Sulfated EPSs are common in all domains of life and EPSs such as sulphoevernan, chondroitin sulfate, dextran sulfate, heparin, and mannan sulfate may enable protection of eukaryotic cells from viruses, including human immunodeficiency virus type 1, by inhibiting virus particle adsorption to host cells [46]. In addition, *T. litoralis* displayed biofilm formation on hydrophilic surfaces under a variety of conditions and, particularly, cell adhesion reached high levels in culture media emended with maltose and/or yeast extract. Indeed, polysaccharide levels in biofilms formed under different growth conditions were similar. The adhesion of marine bacteria to surfaces - a process in which EPSs are likely to play a leading role - is not unusual. However, this occurrence has not been investigated to any extent in hyperthermophilic archaea, though an increasing number of organisms are isolated from hydrothermal vent sites. Further study regarding the EPS productions could display new insights on the natural growth of these organisms providing information on essential ecological interactions in microbial world [47].

**Figure 2:**
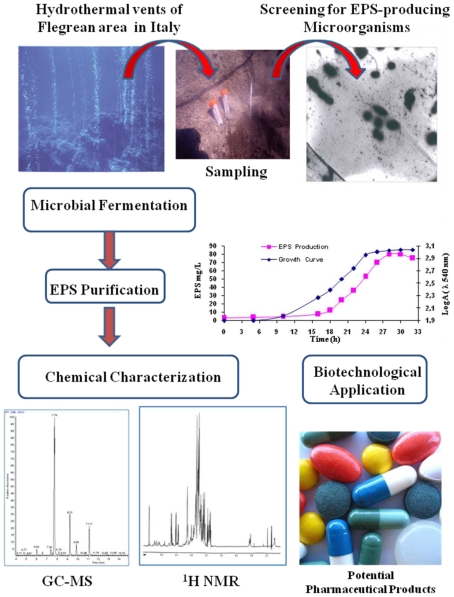
Schematic steps involved in the studies of *Geobacillus* strain 4004 EPS [48].

Additionally, thermophilic bacteria belonging to *Geobacillus* species have been isolated from shallow, marine hydrothermal vents of flegrean area in Italy and characterized as EPS producers as well [48]. Accordingly, a thermophilic strain from the genus *Geobacillus* was isolated from sediment samples in a marine hot spring near the seashore of Maronti (Ischia Island, Italy) and the EPS produced was characterized (Figure 2).

Among different strains isolated, the strain *Geobacillus* sp. 4004 was able to produce an EPS in yields of 90 mg/L in Bacto Marine Broth 2216 (Difco) at the optimal temperature of 60 °C at pH 7.0. Additionally, it was established that, by modulating the conditions of growth as well as emending the culture medium with different carbon sources, the yield of the biopolymer increased and the biomass production was directly proportional to EPS production. Therefore, the EPS produced by strain 4004 in the sucrose medium was isolated via ethanol precipitation of the cell-free medium. The polysaccharide fraction, tested for carbohydrate (85%), protein (10%), and nucleic acid (2%) content, was purified by gel filtration chromatography. The average molecular mass of the strain 4004 EPS was determined to be about 1 × 10^6^ Da and the ^1^H and ^13^C-NMR spectra showed that there are five different residues within the repeating saccharidic unit; two of them with a *gluco/galacto* configuration and three with a *manno* configuration. One of the residues is an acetamido-sugar and at least one uronic acid is present. Sugar analysis was performed on hydrolysed polysaccharide fraction and the EPS was reported to be composed of Gal/Man/GlcN/Ara in a relative ratio of 1.0:0.8:0.4:0.2, respectively. The successful application of EPS largely depends on their physicochemical properties rather than on yield alone. Therefore, in order to gain insight about the viscometric properties, specific viscosity (η) was studied with an Ubbelohde type viscometer under different water concentrations of EPS, in particular at 1% CaCl_2_ and 1% NaCl. It was observed that at increasing concentration of EPS there was an increase in viscosity in all the solutions and the maximum viscosity was found to be 1.08 dL/g in 1% CaCl_2_.

Furthermore, marine shallow hydrothermal vents around volcanic Eolian islands, close to Sicily coasts (Italy), represent accessible fields for the isolation of thermophilic bacteria. Previous studies described diversity and distribution of bacterial communities within deep and shallow hydrothermal systems at Porto di Levante, Vulcano, revealing the presence of chemosynthetic, thermophilic, archaeal and bacterial strains [49]. In addition, a thermophilic aerobic microorganism, able to produce two exocellular polysaccharides (EPS1 and EPS2), was isolated from sea water of a shallow hydrothermal vent at Vulcano island (Eolian Islands, Italy). This new eolian thermophilic isolate was identified as *Bacillus thermodenitrificans* strain B3-72. This strain displayed the highest EPS production in a Bacto Marine Broth 2216 (Difco) at an optimal temperature of 65 °C and pH 7.0 in aerobic conditions. The production started at the end of the exponential phase of growth and continued during the stationary phase. The highest concentrations of polysaccharide harvested after three days of culture was approximately of 70 mg/L. In particular, EPS2, the polymeric fraction obtained after DEAE Gel filtration (80% carbohydrate and 3% proteins content), displayed a molecular weight of approximately 4.0 × 10^5^ Da. Moreover, the IR spectrum of EPS2 suggested the absence of uronic acid and sulfate residues. Hydrolysed EPS2 from *B. thermodenitrificans* yielded mannose and glucose as principal constituents in a relative ratio of 1:0.2. The ^13^C and ^1^H-NMR spectra of this polymer were performed and it was possible to conclude that it possesses a trisaccharide repeating unit essentially constituted of sugars having a *manno*-*pyranosidic* configuration. In subsequent experiments, the immunomodulatory and antiviral effects of the *B. thermodenitrificans* EPS2 were evaluated, since polysaccharides with high molecular weight have exhibited immunogenic activity so far [50]. *In vitro* studies have demonstrated that sulfated polysaccharides, such as dextran sulfated, have antiviral effects against enveloped viruses such as herpes simplex virus [51]. The sulfated-EPS are known to interfere with the absorption and penetration of viruses into host cell and to inhibit various retroviral reverse transcriptases [52]. It was established that the *B. thermodenitrificans* EPS2 obstructs HSV -2 replication in human peripheral blood mononuclear cells (PBMC). Actually, high levels of IFN-α, IL-12, IFN-γ, TNF-α, IL-18 (IFN, interferons; TNF, tumor necrosis factor; IL, interleukine) were detected in supernatants of EPS-2 treated PBMC and, additionally, this effect was dose-dependent. Those results highlight the potential role of *B. thermodenitrificans* EPS2 toward equilibrating the immune response during viral infection and that the immunological disorders determined by HSV-2 could be partially restored by treatment with EPS [53].

Besides, a thermotolerant *Bacillus licheniformis* strain (B3-15), isolated from water of a shallow, marine hot spring at Vulcano Island (Eolian Islands, Italy), produced an EPS with immuno-modulating properties. This strain exhibited a production of 165 mg/L exocellular polysaccharide when grown in liquid mineral medium with the addition of glucose. Furthermore, *Bacillus licheniformis* strain B3-15 showed a high growth rate in media containing kerosene as sole carbon source. The purified exopolymers fraction displayed a single fraction with high carbohydrate content. According to the chemical structure analyses, the EPS was a tetrasaccharide repeating unit essentially constituted by sugars having a *manno-pyranosidic* configuration [35]. Solutions of the *Bacillus licheniformis* EPS added to *in vitro* cultures of human peripheral blood mononuclear cells (PBMC) exhibited a marked, dose-dependent decrease in HSV-2 (Herpes simplex virus type 2) replication [54]. In order to assess whether the antiviral activity induced by EPS in PBMC could be related to an immune-modulatory mechanism, the production of different cytokines involved in the immune response toward virus infection, such as IFN-a, IL-12, IFN-g, TNF-a, IL-18, was evaluated. As a result, high levels of all these cytokines were detected in supernatants from PBMC treated with EPS. On the other hand, IL-4, a strong hallmark of Th2 responses, was not detected in any of the supernatants tested. According to the data collected, the effect of EPS was dose dependent when PBMC were treated with EPS and simultaneously infected with HSV-2 and cytokine production was down-regulated. Consequently, those data suggest that EPS may contribute to improve immune surveillance of PBMC toward virus infection eliciting a therapeutic Th1-like response in clinical settings of viral diseases as well as in immune-compromised host. Actually, a large number of immune-modulatory compounds have been developed in recent years in order to enhance the response of the host to invading infectious agents. However, the accomplishment of such therapeutic efforts relies upon the complete knowledge of cell types and factors involved in the development of a protective antiviral response [54].

### 4.2. Cold Marine Environments: Deep-sea, Arctic and Antarctic Sea Ice

Psychrophilic (“cold loving”) bacteria prefer a growth temperature of less than 15 °C. Bacteria that can grow at such cold temperatures, but which prefer a high growth temperature, are known as psychrotrophs or psychrotolerants.

It has become widely accepted that bacteria isolated from deep-sea vents will represent a valuable resource of unusual molecules for biotechnological purpose and at the same time provide insight into the role of the peculiar molecules produced (Table 2). Most deep-sea environments are influenced by high pressure, low temperature and low nutrient concentration. However, many kinds of deep-sea psychrotolerant bacteria live in the abyssal ecological community and they have been largely investigated as they may reveal important ecological characteristics enabling the morphological, physiological and metabolic adaptation of bacteria to the continuously changing deep-sea ecosystem [55].

*Pseudoalteromonas* sp. SM9913 is a gamma-proteobacterium isolated from 1855 m deep-sea sediment in the Bohai Gulf, the innermost gulf of the Yellow Sea on the coast of north eastern China. Qin *et al.* [56] studied the EPS producing capacity in well established laboratory conditions and reported that the yield of the EPS increased at decreasing culture temperatures in the range of 30–10 °C, and it reached a yield 5.25 g/L (dry weight) under optimal growth conditions (15 °C, 52 h). This is a very high yield compared with the data reported for the EPSs produced by other psychrotolerant microorganisms [57]. Its structure is a linear arrangement of ;-(1→6) linkage of glucose with a high degree of acetylation and with a molecular mass of 4 × 10^4^ Da. It was established that glucose is the main sugar component (61.8%) with minor monosaccharide units including terminal arabinofuranosyl (t-Ara f, 11.0%) and terminal glucopyranosyl (t-Glc, 11.2%) as well as a small amount of t-Gal (3.1%), 4-Xyl f (3.9%), 4-Glc (5.0%) and 3,6-Glc (4.0%). Furthermore, this EPS has been investigated for its flocculation behavior and bio-sorption capacity, providing insight into its ecological rule [58]. Particularly, this polyanionic polymers with high levels of acetyl groups has been shown to bind a wide range of metal cations, such as Fe^2+^, Zn^2+^, Cu^2+^ and Co^2+^, indicating an helpful role in concentrating metal ions to the surface of the strain in the microenvironment around the cell.

Besides, EPSs have a leading role for the survival of psychrophile microorganisms in permanently cold environments. In line with their capacity to protect cells from freezing, high concentrations of EPSs have been found in Antarctic marine bacteria [19] and in Arctic winter sea ice [14]. It has been demonstrated that EPSs produced by sea-ice isolates possess a molecular weight 5–50 times larger than the average of the other isolated marine EPSs and it ranges between 1–3 × 10^5^ Da [5]. The structure and physicochemical properties of the EPSs are influenced by the length of the polymer chain. As the length of the polymer increases, the opportunity for complex entanglement of the chains and intra-molecular associations increases, and these contribute to the tertiary structure and physical behavior of the polymer [22].

It is very interesting to note that particulate aggregates are ubiquitous and abundant in the world’s oceans. Marine bacteria benefit from living in aggregates since their proximity to other cells and surfaces provides opportunities for interaction and nutrient uptake. EPSs excreted by bacteria are polymeric substances that offer a network to hold these structures together [59]. Bacteria are found in abundance in the bottom layers of the ice or in brine channels and are often attached to detrital particles or living microalgal cells. Delille and Rosier [60] also suggested that the high numbers of particle associated bacteria found in sea ice may explain observations of underlying water enriched in bacterial biomass relative to the open ocean. More recently, studies of Arctic sea ice in winter showed that active bacteria could be found in brine channels at temperatures as low as −20 °C and that they were particle associated [13]. EPSs may provide a cryoprotectant role in these environments of high salinities and low temperature [14]. According to this kind of investigation, Mancuso Nichols *et al.* [61] studied the EPS production of two different strains isolated from particulate material and melted sea ice collected in the Southern Ocean. In particular, the bacterial strain CAM025 was isolated from particles collected in melted Antarctic sea ice and CAM036 was isolated from particles captured by a plankton net towed through the Ocean. Both the psychrotolerant strains (growth at 4 °C and 25 °C) displayed an enhanced mucoid morphology on marine agar medium supplemented with glucose. According to the results of 16S rDNA gene sequencing and whole cell fatty acid analyses, these two isolates were shown to be closely related to the genus *Pseudoalteromonas*. Both strains have been cultivated in a Marine Broth Medium enriched with glucose in temperatures ranging from 4–20 °C and the EPS yield data suggest that there is a decreased production of EPS at higher temperature (20 °C) for the Antarctic sea ice strain tested; particularly, strain CAM025 was observed to produce 30-fold more EPS at −2 °C and 10 °C (100 mg/g dry cell weight) than at 20 °C. This result supports the proposed assumption that EPS production by psychrotolerant bacteria may play an important role in the sea ice microbial communities since bacterial EPS production in brine channels and, maybe, other deep cold ecosystems, may offer a fence against the environmental extremes experienced by bacterial cells attempting to modify water properties near the cells. Preliminary characterizations showed that the structure of the EPS from CAM025 and CAM036 includes sulfate as well as high levels of uronic acids, such as galacturonic acid, along with acetyl groups. In addition, the EPS from CAM036 was shown by NMR data to include a succinyl group. Monosaccharide composition was estimated to be Glc/GalA/Rha/Gal (1:0.5:0.1:0.08) and GalA/Glc/Man/GalNAc/Ara (1:0.8:0.84:0.36:0.13) for strain CAM025 and CAM036, respectively. These features suggest an overall polyanionic or ‘sticky’ capacity of the EPSs in the marine environment, since at the pH of seawater (pH 8.0) many of the acidic groups present on these polymers are ionized. This ‘stickiness’ is important since these EPS can bind cations, such as Fe^+3^, whose availability in the Southern Ocean is known to limit primary metabolite production as well as for the uptake of other dissolved metals [62]. According to relative monosaccharide molar ratio, those two EPS displayed some similarities in the composition including the presence of the sole acidic sugar, galacturonic acid, in significant proportion, and glucose as one of the major monosaccharide components as well. Nevertheless, further investigations on the ecological role of the Antarctic EPS could provide insight into possible commercial uses of these novel polymers.

Some EPS producing bacteria have been found to colonize either the Antartic or Artic ocean sea ice. Therefore, the study of the EPS production and the optimal growth condition could provide insight into the ecological role of the EPSs. Recently, Marx *et al.* [63] examined the relative effects of temperature, pressure, and salinity on EPS production by a marine and psychrophilic gamma-proteobacterium, *Colwellia psychrerythraea* strain 34H. This flagella-containing microorganism can be found in the persistently cold marine environments including Arctic and Antarctic sea ice. Strain 34H, in particular, was isolated from Arctic marine sediments. It has been exposed that at growth temperatures ranging from −8 to −14 °C, the EPS production rose dramatically. Similarly, at higher pressures of 400 and 600 atm, the EPS production increased noticeably as well as in salinity tests at 10–100 parts per million (at temperatures of both −1 and −5 °C). Extreme environmental conditions hence appeared to stimulate EPS production by this strain. Furthermore, strain 34H recovered best from deep-freezing to −80 °C if first supplemented with a preparation of its own EPS, rather than other cryoprotectants like glycerol, suggesting that the EPS production is both a survival strategy and a source of compounds with potentially novel properties for biotechnological applications.

### 4.3. Hypersaline Marine Environment: Salt Lakes and Marine Salterns

Hypersaline environments are found in a wide variety of aquatic and terrestrial ecosystems. They are inhabited by halotolerant microorganisms as well as halophilic microorganisms (0.5 M and 2.5 M NaCl) and extreme halophiles (up above 2.5 M NaCl). Moderate and extreme halophiles have been isolated not only from hypersaline ecosystems (salt lakes, marine salterns and saline soils) but also from alkaline ecosystems (alkaline lakes). Halophilic microorganisms have developed various biochemical strategies in order to survive in high saline conditions, including compatible solute synthesis to maintain cell structure and function. Their products such as ectoine, bacteriorhodopsins, exopolysaccharides, hydrolases, biosurfactants are noticeably of industrial interest.

Indeed, the genus *Halomonas* has received increasing interest as several species are able to produce significant quantities of EPS with high surface activity and/or rheological properties [64,65]. Intriguing properties of the EPS derived from *Halomonas* species, such as emulsification activity, appear to be worthwhile for an ample range of products and application. Actually, large quantities of surface-active agents, indispensable components in the food, cosmetic and pharmaceutical sector, are synthesized from hydrocarbons and this represents a problem because they are derived from a non renewable resource arousing concerns about their environmental impact and potential health risks. Conversely, biosurfactants and bioemulsifiers of biological origin have received increasing interest since they are renewable and less toxic compounds. Microbial production of surface-active agents offers a sustainable and potentially cost-effective alternative to chemical synthesis [66]. In addition, according to recent developments in industrial processing technologies, there is an increasing demand for new types of biopolymers with novel or enhanced functionalities and new microbial isolates, particularly of a marine origin, offer a relatively underexploited resource [67].

However, the first EPS to be described was produced by the extremely halophilic archaea *Haloferax mediterranei* (Table 3), isolated from the Mediterranean Sea [68]. In laboratory conditions, the cells were detected to be surrounded by an amorphous matrix in unshaken cultures. In addition, it was recognized, by means of electron microscopy using thin sections and negative staining, a thick layer of EPS surrounds the cells. The EPS production was scarcely influenced by the growth conditions studied and, although the amount of polymer produced with yeast extract or glucose was not strikingly different; the aggregation of the cells on top in unshaken cultures appeared to be dependent on the presence of glucose. This EPS was obtained from the supernatant of shaken liquid cultures by cold ethanol precipitation, and yielded 3 mg/mL. Three neutral sugars were detected such as glucose, galactose, and mannose as major component. The IR spectrum of the EPS indicated the presence of sulfate groups as well as carboxyl group of sterified organic acids such as uronic acids, indicating that the EPS produced is an acidic heteropolysaccaride. The study of rheological properties displayed a pseudo plastic behavior as the EPS solutions indicated a high apparent viscosity, increasing noticeably with the concentration, but the viscosity remained quite constant over wide ranges of pH, temperature and different salinities. Indeed, the extreme salt tolerance of this polysaccharide and the producer organism showed that this microorganism is a valuable candidate for the recovery of oil, especially in oil deposits with high salinity concentrations. In subsequent experiments, Parolis *et al.* [69] elucidated completely the structure of the repeating unit of the EPS produced by *Haloferax mediterranei* by using a combination of chemical and spectroscopic approaches. The EPS was hydrolysed with 4M trifluoroacetic acid and the hydrolysate was converted into alditol acetates to be analysed by GLC. GLC analysis showed only mannitol hexacetate, indicating that mannose was the only neutral sugar present in the polymer. A further portion of the EPS was methanolysed, then the methyl esters were reduced and the products were hydrolysed and converted into alditol acetates as before. GLC analysis showed mannitol hexaacetate and 2-acetamido-2-deoxyglucitol pentaacetate in the molar ratio 1.0:1.1, respectively, indicating that the acidic sugar present in the EPS is 2-amino-2-deoxyglucuronic acid. 1D and 2D NMR spectroscopic analysis of the native and periodate-oxidized/reduced polysaccharide provided the complete structure of repeating unit of EPS as: →4)-β-d-Glc*p*NAcA-(1→6)-α-d-Man*p*-(1→4)-β-d-Glc*p*NAcA-3-*O*-SO_3_^−^-(1→.

A novel EPS, called EPS-R, produced by a slightly halophilic marine bacterium *Hahella chejuensis*, was isolated from a marine sediment sample collected from Marado, Cheju Island, Republic of Korea [28] and displayed a specific emulsifying capacity higher than that observed in commercial polysaccharides such as xanthan gum, gellan gum or sodium alginate. EPS-R contains glucose and galactose as the main sugars components in a molar ratio of 0.68:1.0, respectively; traces of minor sugar components such as xylose and ribose have also been found. The average molecular mass, as determined by gel filtration chromatography, is 2.2 × 10^3^ KDa. The rheological behavior of EPS-R indicated that this EPS formed a structure intermediate between a random-coil polysaccharide and a weak gel. Furthermore, EPS-R was stable to pH and salts, demonstrating proper capacity for future applications as biosurfactant which may enhance hydrophobic substrate utilization and detoxification of polluted areas from petrochemical oils. In fact, biosurfactants from microorganisms are worthwhile more than their chemical counterparts as they are biodegradable and can be synthesized under user-friendly conditions (e.g., low temperatures and pressures) and are effective over a wide range of temperature, pH and salinity conditions [70].

A new halo-alkalophilic *Halomonas* strain has been shown to produce EPS [29,30] The new strain, *Halomonas alkaliantarctica* strain CRSS, was isolated from salt sediments near the salt lake in Cape Russell in Antarctica. The strain CRSS grew aerobically in a complex medium containing 100 g/L NaCl and, among different carbon sources tested, acetate was established to be the most efficient one displaying a yield of 2.9 g/g dry cells at 30 °C after 48 h of growth. The cell-free supernatant was precipated with ethanol and the soluble fraction was further investigated. The hydrolysed EPS produced by strain CRSS presented a monosaccharide composition of Glc/Fru/GlcN/GalN in relative proportion of 1.0:0.7:0.3:tr, respectively. The ^1^H and ^13^C-NMR spectra showed the presence of six different residues in the repetitive saccharidic unit, five of them with α configuration and one with a *manno* configuration indicating a complex primary structure of the biopolymer. Furthermore, in accordance with the measurements of viscosity (η) performed on aqueous solutions of the polysaccharide (1% w/v), an increase of solution viscosity at 2.0–3.0 pH values and at 2.5% (w/v) NaCl was detected according to the size and number of macromolecules in solution [29].

### 4.4. Polychaete Annelid in Symbiotic Relationships

Mutually beneficial symbiotic relationships are widespread in marine ecosystems and complex positive interactions have been shown to be common especially in extreme marine conditions such as hydrothermal and deep sea vents. Recent insights have noticeably expanded the ecological niche biodiversity and increase the number of microorganisms involved in a variety of systems. Accordingly, some marine annelid worms have completely lost the digestive tract of their relatives (the common earthworm) and it has been established that some species get their sustenance from a large population of at least five different species of bacteria living underneath their outer skin. Particularly a polychaete annelid, *Alvinella pompejana*, commonly known as the Pompeii worm, resides in tubes near hydrothermal vents along the seafloor. While in the tube, the worm’s tail end might be immersed in temperatures as hot as 81 °C, while its head rests in cooler water, as moderate as 22 °C. Living in a symbiotic relationship, the worms secrete mucous from tiny glands on their backs to feed the bacteria, and in return they are protected by some degree of insulation. Besides the temperatures, the Pompeii worm has to deal with lethal chemicals too, like sulfides and heavy metals such as lead, cadmium, zinc, and copper. It has been supposed that symbiotic bacteria making the worm’s back their home may detoxify the water within the worm’s tube. Therefore, those bacteria could prove useful in cleaning up toxic waste sites. An EPS-producing strain, designated as HYD657 (Table 4), isolated from epidermis of a polychaete annelid, *Alvinella pompejana,* was collected near an active hydrothermal vent of the East Pacific Rise [71]. Phylogenetic analyses showed that strain HYD657 belonged to the gamma-subdivision of the phylum *Proteobacteria* and that it was closely related to *Alteromonas macleodii*. According to the measurement of DNA-DNA homology, the name of *A. macleodii* subsp. *fijiensis* biovar deepsane was assigned to the bacterium strain HYD657. The monosaccharide composition of the polysaccharide excreted was determined by means of GC, analysing monosaccharide derivates as trimethylsilyl glycosides following methanolysis, and it presented Gal/Glc/Rha/Fuc/Man/GlcA/GalA/3-0-(1 carboxyethyl)-d-GlcA, an unusual diacidic hexose found only in another EPS excreted by a bacterial strain originating from deep-sea vents [72] in a molar ratio of 1:0.42:0.85:0.5:0.42:0.5:0.5:0.5, respectively. Preliminary structural studies on the sugar derivatives by Nuclear Magnetic Resonance and GC-Mass Spectrometry revealed the repeating unit of this polysaccharide was an undesaccharide with three side-chains. Although the definitive structure determination of the polymer requires additional studies, this EPS has been shown to possess interesting biological activities and applications have already been found in cosmetics (patentPCT 94907582-4).

Furthermore, a new facultative anaerobic, heterotrophic and mesophilic bacterium was also isolated from Pompei worm tube collected from a deep-sea hydrothermal field of the East Pacific Rise and named *Vibrio diabolicus* [75]. During the stationary phase of growth in batch cultures on a medium enriched with glucose, *V. diabolicus* strain HE800 produced an EPS characterized by equal amounts of uronic acid and hexosamines (N-acetyl glucosamine and N-acetyl galactosamine) in a molar ratio of approximately 1:0.5:0.5, respectively. Structural studies have recently been conducted on this polymer demonstrating that it consists of a linear tetrasaccharide repeating unit [76]. This novel bacterial polysaccharide has been recently investigated as a bone regeneration and cicatrizing material and a patent has been obtained (patent US 7015206B2). As a result, the EPS excreted by *V. diabolicus* make it possible to fill critical size bone defects in rat calvaria. It can form an attractive extracellular matrix for the direct adhesion of osteoblasts, osteoprogenitor cells and pericytes and may protect the growth and hormonal factors associated with the healing process. The high binding capacity of calcium by this polysaccharide can also be another important parameter in its efficiency to induce fast bone healing. Moreover, it was established that this EPS did not cause any inflammatory reaction and it is completely absorbed by the surrounding tissues. Therefore, this EPS constitutes a material which potentiates bone repair and its particular activity is probably due to its original physicochemical characteristics [76].

*Alteromonas infernus*, strain GY785 was isolated from a sample of fluid collected among a dense population of *Riftia pachyptila* in the proximity of an active hydrothermal vent of the Southern depression of the Guaymas basin (Gulf of California). This strain, in a glucose enriched medium, secreted a water-soluble acidic heteropolysaccharide consisting of glucose, galactose, glucuronic and galacturonic acids (1:1:0.7:0.4), respectively. This high-molecular-weight polysaccharide (1 × 10^6^ Da) differed in monosaccharide content and/or ratio and sulfate content (10%) from other EPS isolated from deep-sea hydrothermal bacteria. Different fractions of the purified EPS have been chemically modified by sulfation and acidic depolymerization leading to an elevated increase of the attached sulfated groups (33–40%) producing low-molecular-weight, low-viscous EPSs in yields ranging from 25–50% [43]. In addition, the overall monosaccharide composition of all the fractions is not chemical modified by those reactions and the high sulfate content up to 40% of the low-molecular-weight fractions along with the nature of the sugar units should give rise to original bioactive compounds. However, regioselectivity of the reaction is an important issue as the biological activity can be related to the position of the sulfate groups in the polymers as it is reported for other highly sulfated polysaccharides, such as chondroitin sulfate, mannans, dermatan sulfates, fucoidan [77,78] k-carrageenan and heparin [79], which have been shown to possess important physiological functions and they have been applied as therapeutic anticoagulant and antithrombotic agents. Moreover, the original backbone structure of *A. infernus* EPS is made up primarily of glucose, galactose and uronic acid residues bound by→13 or/and 1→4 linkages, in the manner of most other anticoagulant 1 polysaccharides. Original sulfation patterns were obtained due to this unique backbone structure consisting of many different monosaccharide building units, glycosidic linkage types and unit branches. Consequently, this kind of marine EPS can be used as a library of compounds useful for the elucidation of the structure-activity relationship and would represent a valid alternative to mammalian sources of anticoagulant avoiding the risk of contamination with pathogenic agents potentially present in mammalian tissues [43].

## 5. Conclusion

Microbial EPSs are ubiquitous in the extreme marine environment where they are essential for microbial survival. Most of the functions ascribed to EPS are of a protective nature and their precise roles are dependent on the ecological niches in which the microorganisms live. They could assist the microbial communities to endure extremes of temperature, salinity and nutrient availability, creating a boundary between the bacterial cell and its immediate environment. Several EPSs produced by microorganisms from extreme habitats show biotechnological promise. By examining their structure and chemical-physical characteristics, it is possible to gain insight into their commercial application and they are employed in several industries ranging from pharmaceutical to food-processing fields, through to the detoxification capability of polluted areas from petrochemical oils.

Considering that the microbial biodiversity of marine eco-systems is relatively unexplored, it is reasonable to hypothesize that the isolation and identification of new microorganisms will provide wide opportunities for new industrial fields.

## Figures and Tables

**Table 1:** EPSs produced by microorganisms isolated from marine hot springs and hydrothermal vents.

Microorganisms	Source Environment	Description of EPS and Chemical Composition	Suggested Ecological Role and Biotechnological Application	References
*Pseudoalteromonas* strain 721	Deep-sea hydrothermal vent	Octasaccharide repeating unit with two side chains, (Figure 1)	Gelling properties	[39,40]
*Alteromonas macleodii* subsp. *fijiensis*	Deep-sea hydrothermal vent, North Fijian Basin	Sulfated heteropolysaccharide, high uronic acids with pyruvate. The repeating unit is a branched hexasaccharide containing Glc, Man, Gal, GlcA, GalA, pyruvated mannose	Thickening agent in food- processing industry, biotoxification and waste- water treatment, bone healing, treatment of cardiovascular diseases	[16,17,37,41–43]
*Thermococcus litoralis*	Shallow submarine thermal spring	Man is the only monosaccharide	Biofilm formation	[47]
*Geobacillus* sp. strain 4004	Sediment in marine hot spring near the seashore of Maronti, Ischia Island, Italy	A pentasaccharide repeating unit (two of them with a gluco-galacto configuration and three with a *manno* configuration. Gal:Man:GlcN:Arab (1.0:0.8:0.4:02)	Pharmaceutical application	[48]
*Bacillus thermodenitrificans* strain B3-72	Water of a shallow hydrothermal vent, Vulcano Island, Italy	Trisaccharide repeating unit and a *mannopyranosidic* configuration. Man:Glc (1:0.2)	Immunomodulatory and antiviral activities	[49,53]
*Bacillus licheniformis* strain B3-15	Water of a shallow marine hot spring, Vulcano Island, Italy	Man is the main monosaccharide. Tetrasaccharide repeating unit and a *mannopyranosidic* configuration	Antiviral activity	[35,54]

**Table 2:** EPSs produced by microorganisms isolated from cold marine environments.

Microorganisms	Source environment	Description of EPS and Chemical composition	Suggested Ecological Role and Biotechnological Application	References
*Pseudoalteromonas* strain SM9913	Deep-sea sediment in the Bohai Gulf, gulf of the Yellow Sea, China	Linear arrangement of α-(1→6) linkage of glucose with a high degree of acetylation	Flocculation behavior and bio-sorption capacity	[56,58]
*Pseudoalteromonas* strain CAM025	Isolated from particles collected in melted Antarctic sea	Sulfated heteropolysaccharide, high levels of uronic acids with acetyl groups Glc:GalA:Rha:Gal (1:0.5:0.1:0.08)	Cryoprotection	[61]
*Pseudoalteromonas* strain CAM036	Isolated from particles captured by a plankton net towed through the Southern Ocean	Sulfated heteropolysaccharide, high levels of uronic acids with acetyl and succinyl groups GalA:Glc:Man:GalNAc:Ara (1:0.8:0.84:0.36:0.13)	Trace metal binding	[61]
*Colwellia psychrerythraea* strain 34H	Arctic marine sediments	n.r.	Cryoprotection	[63]

n.r. not reported.

**Table 3:** EPSs produced by microorganisms isolated from hypersaline marine environments.

Microorganisms	Source environment	Description of EPS and Chemical composition	Suggested ecological role and Potential Biotechnological Application	References
*Haloferax mediterranei*	Mediterranean Sea	→4)-β-D-Glc*p*NAcA- (1→6)-α-D-Man*p*- (1→4)-β-D-Glc*p*NAcA- 3-*O*-SO_3_--(1→	Candidate in oil recovery, especially in oil deposits with high salinity concentrations	[68,69]
*Hahella chejuensis*	Marine sediment sample collected from Marado, Cheju Island, Republic of Korea	EPS named EPS-R Glc:Gal (0.68:1.0)	Biosurfactant and detoxification of polluted areas from petrochemical oils	[28]
*Halomonas alkaliantarctica* strain CRSS	Salt lake in Cape Russell in Antarctica	Glc:Fru:GlcN:GalN (1.0:0.7:0.3:trace)	High viscosity	[29,30]

**Table 4:** EPSs produced by marine microorganisms involved in symbiotic relationships.

Microorganisms	Source environment	Description of EPS and Chemical composition	Suggested ecological role and Potential Biotechnological Application	References
*Alteromonas macleodii* subsp. *fijiensis* biovar deepsane strain HYD657	Isolated from epidermis of a polychaete annelid, *Alvinella pompejana*, hydrothermal vent of the East Pacific Rise	The repeating unit is an undesaccharide with three side-chains. Gal:Glc:Rha:Fuc:Man:GlcA:GalA:3-0-(1 carboxyethyl)-D-GlcA (1:0.42:0.85:0.5:0.42:0.5:0.5:0.5)	Cosmetics (patent PCT 94907582-4)	[71]
*Alteromonas* strain 1644	Isolated from *Alvinellidae* collected near hydrothermal vent of the East Pacific Rise	Main chain of five sugars with a side chain of three sugars including a dicarboxylic acid. Glc:Gal:GlcA:3Lac-GlcA:GalA	Heavy metal binding	[73,74]
*Vibrio diabolicus* strain HE800	Isolated from a Pompei worm tube collected from a deep-sea hydrothermal field of the East Pacific Rise	A linear tetrasaccharide repeating unit. Uronic acid :GlcN:GalN 1:0.5:0.5 →3)-β-D-Glc*p*NAc-(1→4)-β-D-Glc*p*A- (1→4)-β-D-Glc*p*A-(1→4)-α-D-Gal*p*NAc- (1→	Bone regeneration and cicatrizing material (patent US 7015206B2)	[75,76,80,81]
*Alteromonas infernus* strain 785	Isolated from a fluid sample collected among a dense population of *Riftia pachyptila* in the proximity of an active hydrothermal vent, Guaymas basin (Gulf of California)	Glc:Gal:GlcA:GalA (1:1:0.7:0.4)	Anticoagulant activity	[43]
